# Lung cancer in the emergency department

**DOI:** 10.1186/s44201-023-00018-9

**Published:** 2023-03-06

**Authors:** Jeremy R. Walder, Saadia A. Faiz, Marcelo Sandoval

**Affiliations:** 1 Divisions of Critical Care, Pulmonary and Sleep Medicine, McGovern Medical School at UTHealth, 6431 Fannin St., Ste. MSB 1.282, Houston, TX 77030 USA; 2 Department of Pulmonary Medicine, The University of Texas MD Anderson Cancer Center, 1515 Holcombe Blvd., Unit 1462, Houston, TX 77030 USA; 3 Department of Emergency Medicine, The University of Texas MD Anderson Cancer Center, 1515 Holcombe Blvd., Unit 1468, Houston, TX 77030 USA

**Keywords:** Lung cancer, Emergency department, Lung cancer screening

## Abstract

**Background:**

Though decreasing in incidence and mortality in the USA, lung cancer remains the deadliest of all cancers. For a significant number of patients, the emergency department (ED) provides the first pivotal step in lung cancer prevention, diagnosis, and management. As screening recommendations and treatments advance, ED providers must stay up-to-date with the latest lung cancer recommendations. The purpose of this review is to identify the many ways that emergency providers may intersect with the disease spectrum of lung cancer and provide an updated array of knowledge regarding detection, management, complications, and interdisciplinary care.

**Findings:**

Lung cancer, encompassing 10–12% of cancer-related emergency department visits and a 66% admission rate, is the most fatal malignancy in both men and women. Most patients presenting to the ED have not seen a primary care provider or undergone screening. Ultimately, half of those with a new lung cancer diagnosis in the ED die within 1 year. Incidental findings on computed tomography are mostly benign, but emergency staff must be aware of the factors that make them high risk. Radiologic presentations range from asymptomatic nodules to diffuse metastatic lesions with predominately pulmonary symptoms, and some may present with extra-thoracic manifestations including neurologic. The short-term prognosis for ED lung cancer patients is worse than that of other malignancies. Screening offers new hope through earlier diagnosis but is underutilized which may be due to racial and socioeconomic disparities. New treatments provide optimism but lead to new complications, some long-term. Multidisciplinary care is essential, and emergency medicine is responsible for the disposition of patients to the appropriate specialists at inpatient and outpatient centers.

**Conclusion:**

ED providers are intimately involved in all aspects of lung cancer care. Risk factor modification and referral for lung cancer screening are opportunities to further enhance patient care. In addition, with the advent of newer cancer therapies, ED providers must stay vigilant and up-to-date with all aspects of lung cancer including disparities, staging, symptoms of disease, prognosis, treatment, and therapy-related complications.

## Background

**Fig. 1:**
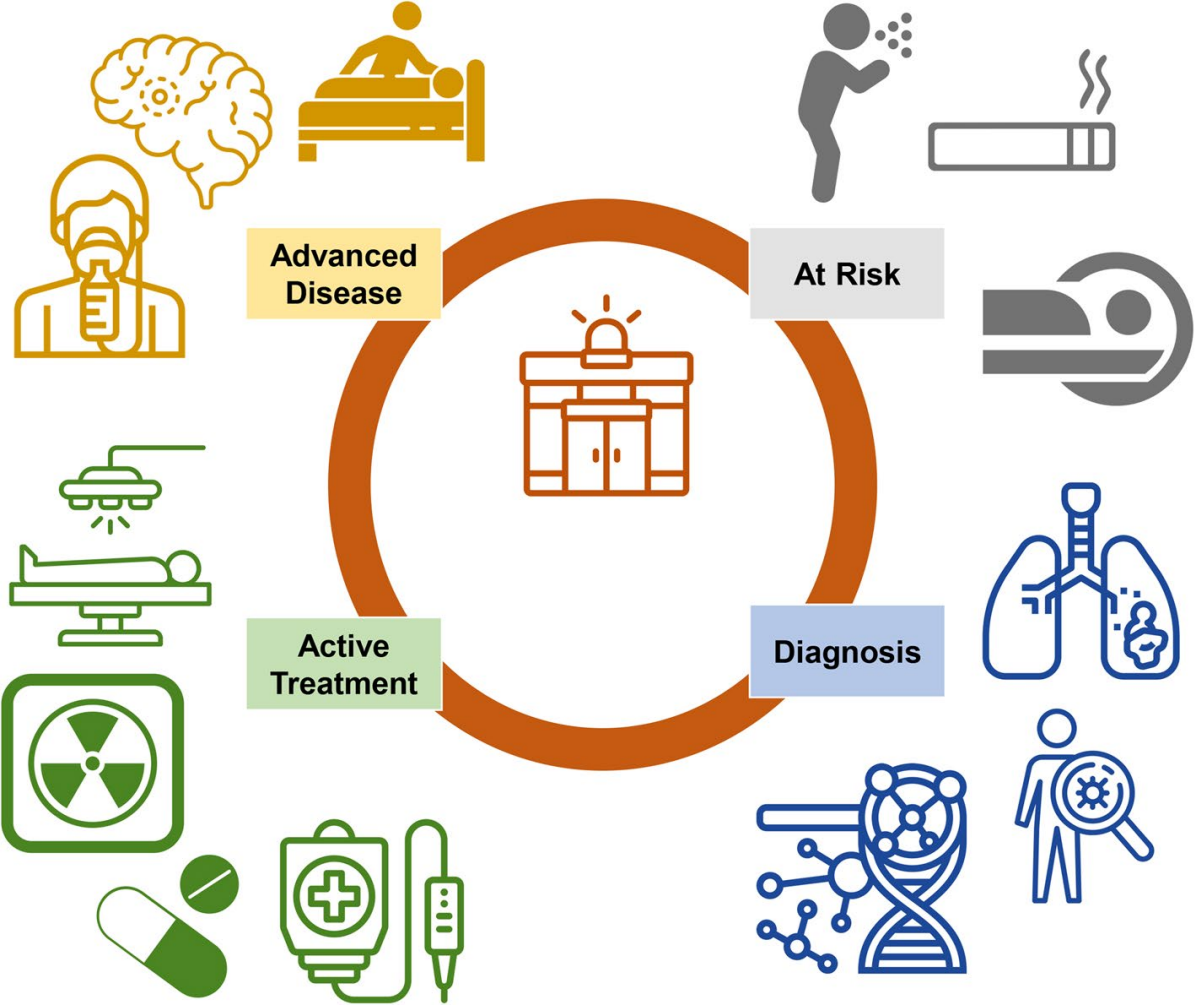
Lung cancer and emergency department. ED providers may encounter lung cancer along the cancer continuum. The ED may play a role in identifying those at risk of developing lung cancer or referring for further screening those with concerning findings. Incidental lung cancer may present with or without symptoms, and appropriate staging and tissue diagnosis with molecular markers are crucial. Sequelae from active treatment may include pain, radiation-induced lung injury, or drug-related pneumonitis. In those with advanced or metastatic disease, stabilization, symptom management, and supportive care are often required

Emergency department (ED) providers encounter lung cancer patients across the continuum of their malignancy (Fig. 1). Lung cancer engenders a significant symptom burden especially affecting the respiratory system, and it is the leading cause of cancer-related deaths worldwide [1]. Tobacco use is the main risk factor for lung cancer, plus other comorbid conditions, such as cardiac disease and chronic obstructive pulmonary disease (COPD), that may bring the patient to the ED and generate an incidental lung cancer diagnosis [2–5]. Furthermore, 40 to 65% of patients with lung cancer will present to the ED at least once during the course of their disease [6, 7]. Thus, it is imperative to recognize symptoms of a malignant disease as well as sequelae from various treatment modalities including chemotherapy, radiation, immunotherapy, and targeted therapies. This review highlights the epidemiology of lung cancer in the ED setting, the importance and potential role of the ED in prevention and screening, and the various clinical intersections of lung cancer and the ED.

## Lung cancer: general information

Lung cancer is the most fatal cancer in both men and women with an estimated 2.2 million new cases and 1.8 million deaths per year worldwide [1]. It is a heterogeneous disease, and it can be classified as non-small cell lung cancer (NSCLC, 85%) or small cell lung cancer (SCLC, 15%). Adenocarcinoma followed by squamous cell carcinoma comprises the most common subtypes of NSCLC. One of the most important predictors of survival is cancer stage at the time of diagnosis, and although localized disease in NSCLC has a 5-year survival of 57.4%, distant metastatic disease 5-year survival is extremely poor at 5.2% [8]. Interestingly, mortality in the United States of America (USA) based on data from Surveillance, Epidemiology, and End Results Program fell sharply from 2013 to 2016, and analysis suggests both reduction in incidence and treatment advances likely explain the trend [9]. Tobacco cessation (to modify risk factor) and lung cancer screening (to detect disease early) are both opportunities where the ED could play a pivotal role. Furthermore, healthcare disparities in high-risk populations including Black individuals [10], those with human immunodeficiency virus (HIV) [11], and socially disadvantaged groups exist, and these groups are disproportionately affected by lung cancer [8]. These populations represent another avenue where the ED can optimize detection, encourage risk factor modification, and potentially facilitate further cancer care.

## Lung cancer in the emergency department

### Epidemiology

Common themes of ED and cancer care have been derived from the Nationwide Emergency Department Sample (NEDS) which is the largest administrative database. Cancer patients comprised 2.4 to 4.2% of the more than 100 million annual ED visits with a 60 to 65% admission rate noted in separate studies [12, 13]. Between 2006 and 2015, hospitals saw a fivefold increase in overall ED visits for oncologic treatment-related complications [14]. Risk factors for ED use include older age, non-White, male, urban-dwelling, low income, Medicaid/uninsured status, comorbidities, history of prior ED use, and end-of-life status [7, 15].

According to statewide and national healthcare utilization databases, lung cancer ranks near or at the top of reasons for cancer-related ED visits encompassing 10 to 12% of cancer-related ED visits, with 66% admission rates and 4.6% in-hospital mortality rates [7, 12, 13, 15–18]. These data reflect its high prevalence and significant morbidity and mortality [19]. Remarkably, in a 2021 single tertiary care center, in those with ED-diagnosed lung cancer, only 16% of patients had seen a primary care provider, and among the 84% eligible, only 6% had undergone screening [20]. Although small studies suggest new lung cancer workup in the ED is expedited, patients tend to have more advanced cancer stages upon presentation [20, 21]. Other sociodemographic features include low income, substance use, and disproportionately increased risk in Blacks (12.4% ED cohort vs 7.9% of the total cohort) [22]. In general, all of these features were associated with higher mortality [23].

Data representing the collective experience in the United States of America (USA) with lung cancer in the ED is limited. A 2022 review of unplanned care revealed that large national ED and hospital administrative databases do not effectively communicate with local–regional cancer registries [7]. The former is rich in International Classification of Diseases codes and demographics but short on cancer staging and treatment information while the latter fails to capture ED utilization data. Efforts to integrate data-rich electronic health records (EHR) with registries are underway but are in the early stages [24].

Literature from other countries provides valuable information about this population. Beckett and colleagues analyzed data from the UK National Lung Cancer Audit from 2006 to 2011 where 19% of patients with lung cancer were diagnosed in the ED. These patients had low socioeconomic status, advanced stage, worse performance status, and higher 1-year mortality [25]. In another study, Elliss-Brookes and associates using national United Kingdom (UK) databases from 2006 to 2008 revealed 39% of lung cancers were diagnosed in the ED and had substantially lower 1-year relative survival [26]. A French study using national databases evaluated 144,087 patients with their first hospitalization for lung cancer from 2016 to 2018; 3-month mortality was 19% and significantly higher for those older than 70, male, metastatic disease at diagnosis, and first hospitalization via the ED [27]. A 2017 systematic review of ED cancer diagnosis studies from developed countries (mostly the UK) found patients were more likely economically deprived, non-White, and non-curable and had lower survival than electively diagnosed counterparts, and lung cancer patients had the highest percentage (59%) of stage 4 presentation among all ED diagnosed cancers [28]. Similar findings regarding advanced disease and mortality in ED-diagnosed patients were seen in subsequent Australian and Canadian studies [29–31]. US studies show race and economic factors play a role in de novo ED lung cancer diagnosis and mortality [20–23]. In contrast, an analysis of 771 patients with advanced NSCLC in Japan where 13% were diagnosed in the ED showed that diagnosis following emergency admission was not an independent predictor of overall survival; however, the following were the independent predictors of overall survival: good performance status, epidermal growth factor receptor (EGFR) receptor status, stage IIIB, adenocarcinoma, and chemotherapy [32]. Interestingly, as the data for each country varies, so does the referral pattern for cancer care. For example, in Japan, patients suspected of lung cancer may directly contact their respiratory physician, whereas in the USA or UK, a referral is needed [33]. In other countries such as Belgium or France, health systems allow patients to choose their physicians directly. These studies all highlight that lung cancer patients present to the ED due to symptoms, often with advanced disease, and interestingly, results appear to vary in part related to the need for referral in different healthcare systems.

### Symptoms

Patients with lung cancer can present to the ED for various reasons, and co-morbid conditions including diabetes, COPD, and vasculopathy often prompt ED visits [18, 34]. Hemoptysis, weight loss, loss of appetite, dyspnea, chest pain, fatigue, and cough were most commonly associated with lung cancer prior to diagnosis in a large population-based case–control study [35]. Neurological concerns (paralysis, seizures, headache, altered consciousness) are common extra-thoracic manifestations. Fujimoto and associates reported lung cancer patients presented in the ED with neurological complaints (23%) and respiratory issues (pleural effusions, 16%; pneumonia, 15%) [32]. In a UK cohort of 269 patients, the main reasons for ED consultation were respiratory symptoms (22.3%), fever (19.9%), and neurological issues (14.2%) [6]. Respiratory symptoms (31.5%) and neurological events (11.2%) represented the first and third most common reasons for ED visits in Japan, respectively [36].

### Radiographic findings

ED patients get thoracic imaging that sometimes reveals unexpected lung lesions. In a single-center cohort, patients who were ultimately diagnosed with lung cancer after ED imaging were more likely to have a concerning finding on computed tomography (CT) versus chest radiograph (CXR) (55.9% vs 36.8%, respectively), and CT was more likely to mention malignancy (OR 5.9, 95% CI 2.5–14.0) or metastasis (OR 30, 95% CI 7.1–12.1). In a few, findings on non-thoracic imaging ultimately led to the diagnosis [20]. Radiographic findings concerning malignancy include lung mass (greater than or equal to 30 mm), pleural effusion or thickening, mediastinal lymphadenopathy, endobronchial lesions, spiculated/lobular contours, post-obstructive pneumonia, ground glass lesions with a solid component, and growth relative to prior imaging [37]. The Fleischner Society provides guidelines for radiographic follow-up of incidental nodules based on patient risk factors, nodule size (< 6 mm low, > 8 mm high), solid or subsolid (ground glass vs part-solid), shape (rounded or irregular), location, number [38].

### Prognosis

Prognosis of lung cancer patients presenting to the ED appears to be worse than other cancer patients. At a single cancer center ED, dyspnea, altered mental status, and diagnosis of lung cancer each independently predicted ICU admission and in-hospital death [39]. A retrospective study of ED intubated cancer patients evaluating 28-day mortality found the highest risk in those with lung cancer compared to non-lung cancer, with the highest being metastatic lung cancer (OR 7.17, 95% CI 2.14–24.01), followed by lung cancer patients without metastases (OR 5.89, 95% CI 1.48–23.36) [40]. A multicenter prospective cohort study of stage IV solid cancer patients in septic shock found lung cancer patients had the highest 28-day mortality at 48.1% [41]. Lastly, several large nationwide hospitalization studies have all found that among cancer patients with sepsis, lung cancer patients were the most likely to die [42–44]. These studies highlight the poor short-term prognosis of severe illness associated with lung cancer. 

### Additional comments

A thorough search was performed using PubMed, but the literature on the intersection of lung cancer and the ED is limited. We provide a summary of data to date, as well as highlighting the results from select studies. Cumulatively, these all provide valuable information, but variability in study design and/or selection bias can limit the results from the various studies. Further large-scale prospective and comparative trials are needed, and the assimilation of various cancer-related healthcare databases could also provide important information in the future.

## Lung cancer screening

Early diagnosis and surgical resection are key to the cure and survival in lung cancer [45, 46]. In the past, CXR and sputum cytology were studied to screen cancer. Due to the overdiagnosis of more early indolent cancers that would never spread, 5-year survival artificially increased, but mortality (deaths per 100,000 per year), the true measure of intervention benefit, did not improve [47]. More recent studies centered around the use of low-dose computed tomography (LDCT). The National Lung Screening Trial (NLST) and the Nederlands-Leuvens Longkanker Screenings Onderzoek (NELSON) trials [48, 49] both provided crucial mortality reduction evidence to support the use of LDCT for lung cancer screening.

### NLST

The NLST recruited 53,454 current or former smokers (who quit less than 15 years ago) with 30 pack-years, aged 55–74, randomized to either 3 annual screening CXRs or LDCTs then followed for 10 years. New nodules/masses ≥ 4 mm were referred for workup. The NLST LDCT used less than 25% of standard CT radiation dosage [50].

For the first time, mortality reduction (20%) from screening was shown. However, it was criticized for overdiagnosis of slow-growing lesions and high rates of false positives resulting in costly and invasive workups [51–54]. In 2014, the American College of Radiology created the Lung CT Screening Reporting & Data System (Lung-RADS®) that recommended increasing the “positive” nodule size threshold to 6 mm and applying evidence-based criteria to their management [55]. If these rules were applied to NLST data, there would have been a 50–75% decrease in false positives and fewer imaging and interventions [56]. Importantly, Lung-RADS® is meant for nodules found on screening, not for nodules found in practice.

### NELSON trial

The Dutch-Belgian NELSON trial intentionally recruited a younger cohort with less intense smoking histories and randomized 13,195 men and 2,594 women to LDCT screening versus standard care. The screening timing was baseline, 1 year, 3 years, and 5.5 years. Unlike NLST, they designed a protocol to minimize repeat scans and invasive testing. NELSON used even lower doses of radiation [57]. Updated scanners and software allowed 3D volumetric nodule measurements. Three nodule categories (negative, indeterminate, and positive) were created based on size and other features [58]. Indeterminate nodules would be re-scanned within 3 months to determine volume doubling time. Positive and rapidly growing indeterminate nodules would be referred for biopsy. Low-risk or slow-growing indeterminate nodules would be recorded but neither biopsied nor rescanned until the next scheduled screening [59]. The results showed a 24% mortality reduction in men and 33% in women; the small sample size made the results in women suggestive but not conclusive. False positives were only 1.2%, as opposed to 24% in NLST putting any doubts about the benefit of LDCT screening to rest [49, 60].

### Additional studies

A 2013 review for the US Preventative Services Task Force (USPSTF) noted the NLST number needed to screen of 320 to prevent 1 death was better than that for breast and colon cancer screening [61]. USPSTF recommended that annual LDCT lung cancer screening be adopted, leading the Centers for Medicare and Medicaid Services (CMS) to approve LDCT screening as a preventative health benefit for all eligible Americans in 2015 [62, 63].

A 2015 National Cancer Institute study found that reducing the smoking requirement for screening to 20 pack-years would include more women and minorities [64]. Another study showed that reducing the screening age threshold to 50 would benefit Black people, who were underrepresented in the NSLT trial (4.4% of trial participants vs 12.3% US population), and were found to develop lung cancer at a younger age and decreased pack-year smoking history compared to the non-Hispanic White population [65]. Since 2012, the National Comprehensive Cancer Network (NCCN) advocated screening certain high-risk younger patients with less smoking history [66]. Due to the NELSON study, their current version 1.2023 guideline’s high-risk category now includes anyone older than 50 with 20 + pack-years [67].

In 2021, the USPSTF broadened their previous 2013 recommendations for lung cancer screening with LDCT to include all current or former smokers aged 50–80 (previously 55–80) who quit less than 15 years ago and 20 pack-year (previously 30) smoking history. The broadened criteria were largely due to the NELSON trial and data from computer modeling studies by the Cancer Intervention and Surveillance Modeling Network, which found superior mortality benefits and life years gained under the new criteria. The expanded criteria would allow for a greater number of women and minority racial or ethnic groups who may develop lung cancer with lower smoking histories. CMS now covers LDCT screening for lung cancer at reduced age (50–77) and smoking (20 pack-years) criteria [68]. There have been calls to augment USPSTF recommendations even further by using risk or benefit model calculators. The American College of Chest Physicians’ guidelines support the use of calculators with screening, for it may lead to greater equity across race and gender by identifying those most likely to benefit [69].

### Barriers and disparities

Lung cancer screening with LDCT scanning can save lives. However, only 4% of those eligible are getting it [70]. Both patient and healthcare provider-related factors can affect screening. The adherence rates are higher in the northeast and lowest in the south [71]. Non-White race, younger age, and current smoking are predictors of non-adherence as is incomplete college education [72–74]. Interestingly, Black people with higher education and eligible for screening adhere more than their White counterparts [75]. Other potential barriers for patients include fear of positive test, radiation exposure, inconvenience, distrust of the medical system, and cost [76, 77]. Patients living in rural or remote areas may have difficulty accessing centers that provide screening and LDCT [78]. Barriers for healthcare providers may include unfamiliarity with screening guidelines, difficulty identifying eligible patients for screening, access to a specialist to address abnormal or equivocal results, skepticism about the benefits, and lack of time or personnel for follow-up [77].

Many have advocated for changes in lung cancer screening programs to promote health equity. A myriad of factors can result in disparities in screening including racial and ethnic background, access to smoking cessation interventions, use of preventive services, and geographical barriers [8]. Marketing for tobacco directed at young adults, minorities (Blacks and non-White Hispanics), and women have directly impacted trends for lung cancer incidence and mortality [1, 79, 80]. Furthermore, screening guidelines center around tobacco use, but 25% of lung cancer cases worldwide are not associated with tobacco use. Globally, lung cancer in never-smokers inherently contributes to gender disparities, as women never-smokers have a high incidence of adenocarcinoma along with unique risk factors for lung cancer [81].

### Role of ED

The role of the ED in lung cancer risk factor modification and screening has garnered attention. EDs can champion smoking cessation programs, initiate referrals, and open conversations [82–84]. EDs have helped enroll patients in colon, prostate, breast, and cervical cancer screening [85–92]. It has been postulated that EDs see many patients who are eligible for lung cancer screening, but often they do not have a primary care provider. A large National Health Interview Survey sample found 25% of patients eligible for lung cancer screening who were non-adherent had visited an ED in the prior year, thus representing an opportunity for outreach [93]. At a single safety net center, though most patients had insurance and access to primary care, no ED patient eligible for lung cancer screening had enrolled, and only 0.06% were aware of lung cancer screening [94]. Comparatively, this is much lower than for colon or breast cancer screening, and it identifies the need for education of patients and providers about lung cancer screening. One effort through California-based EDs using trained associates led to 50% of the intervention group getting screenings within 30 days, including lung cancer [95]. Similar recruitment studies also used trained research associates, implying that dedicated non-clinical staff can successfully identify and motivate patients, relieving the time burden otherwise imposed on busy EDs. Partnerships with local screening efforts will likely result in success. Finally, the ED may be able to play a role in addressing healthcare disparities in lung cancer. Additional counseling on tobacco cessation or referral to preventative services for at-risk individuals can significantly impact risk factors and disease. These grassroots efforts emphasize a valuable opportunity, and translating this into widespread ED-based recruitment is a topic ripe for future research.

## Clinical workup and treatment

### Lung cancer staging

**Fig. 2:**
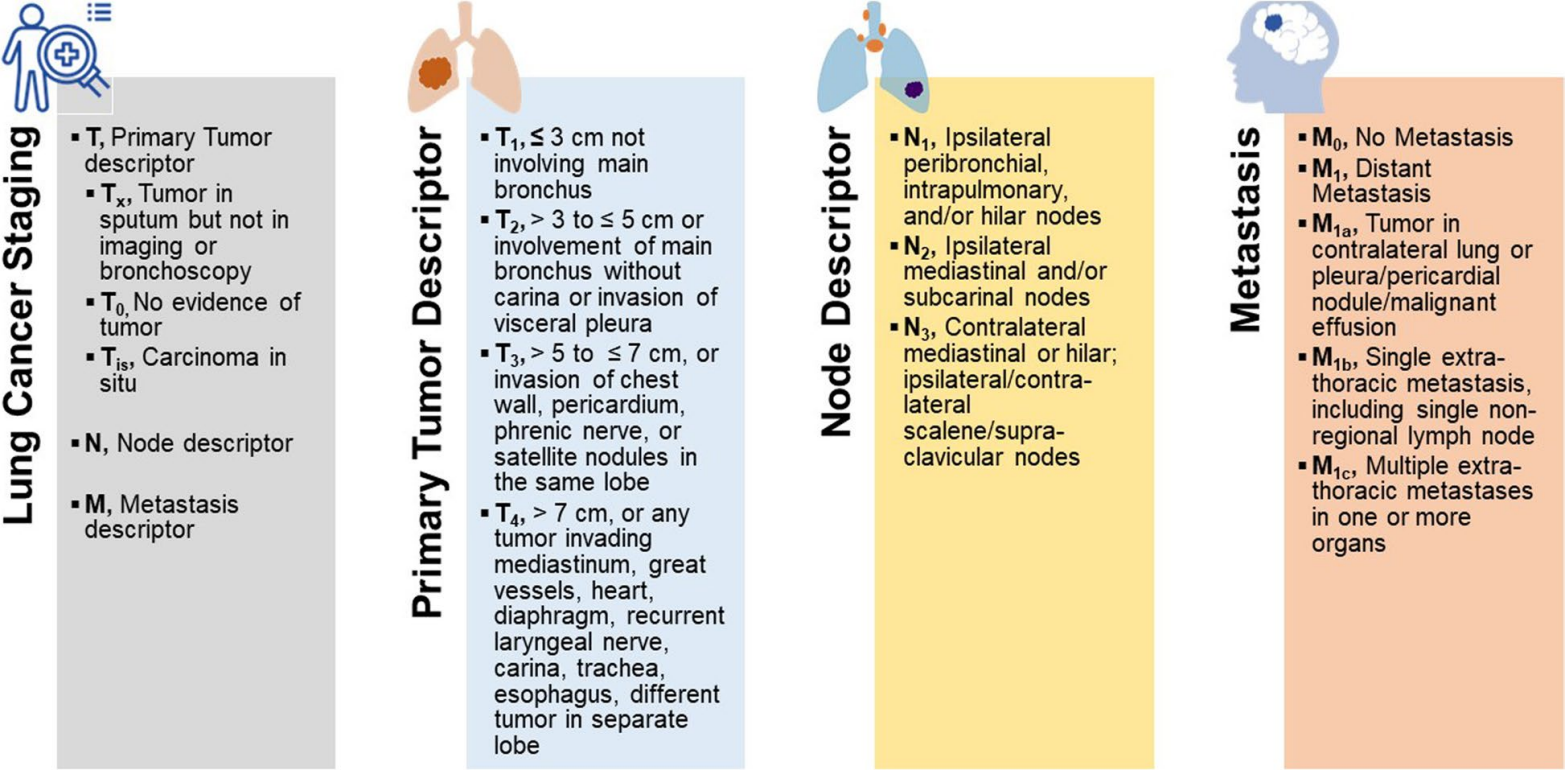
Lung cancer staging. T refers to the primary tumor size and ranges from no primary tumor (T_0_) to a tumor more than 7 cm in the greatest dimension (T_4_). N describes regional lymph node involvement and broadly includes no regional lymph node involvement (N_0_) to contralateral mediastinal, hilar, scalene, or supraclavicular or ipsilateral scalene or supraclavicular lymph node metastasis (N_3_). The presence of distant metastasis is categorized as M_0_ (no distant metastasis) and M_1_ (distant metastasis). M_1_ is further delineated into subclasses where M_1a_ consists of secondary nodules in the contralateral lung, pericardium, or pleura, with or without the presence of a malignant pleural or pericardial effusion. M_1b_ involves one extra-thoracic metastasis, and M_1c_ accounts for multiple extra-thoracic metastases. Information derived from [96]

Staging allows for an unanimously understood categorization system for the treatment and progression of cancer. The American Joint Committee on Cancer (AJCC) incorporates the staging system for both NSCLC and SCLC, and the 8th Edition Lung Cancer Stage Classification is the current internationally recognized system [96] (Fig. 2). Small cell lung cancer (SCLC) is categorized into limited stage and extensive stage. The limited stage incorporates local disease in one radiation field which includes ipsilateral mediastinal or supraclavicular nodal metastases while the extensive stage consists of contralateral or distant metastases and inclusion of any malignant pleural or pericardial effusion [97].

**Fig. 3:**
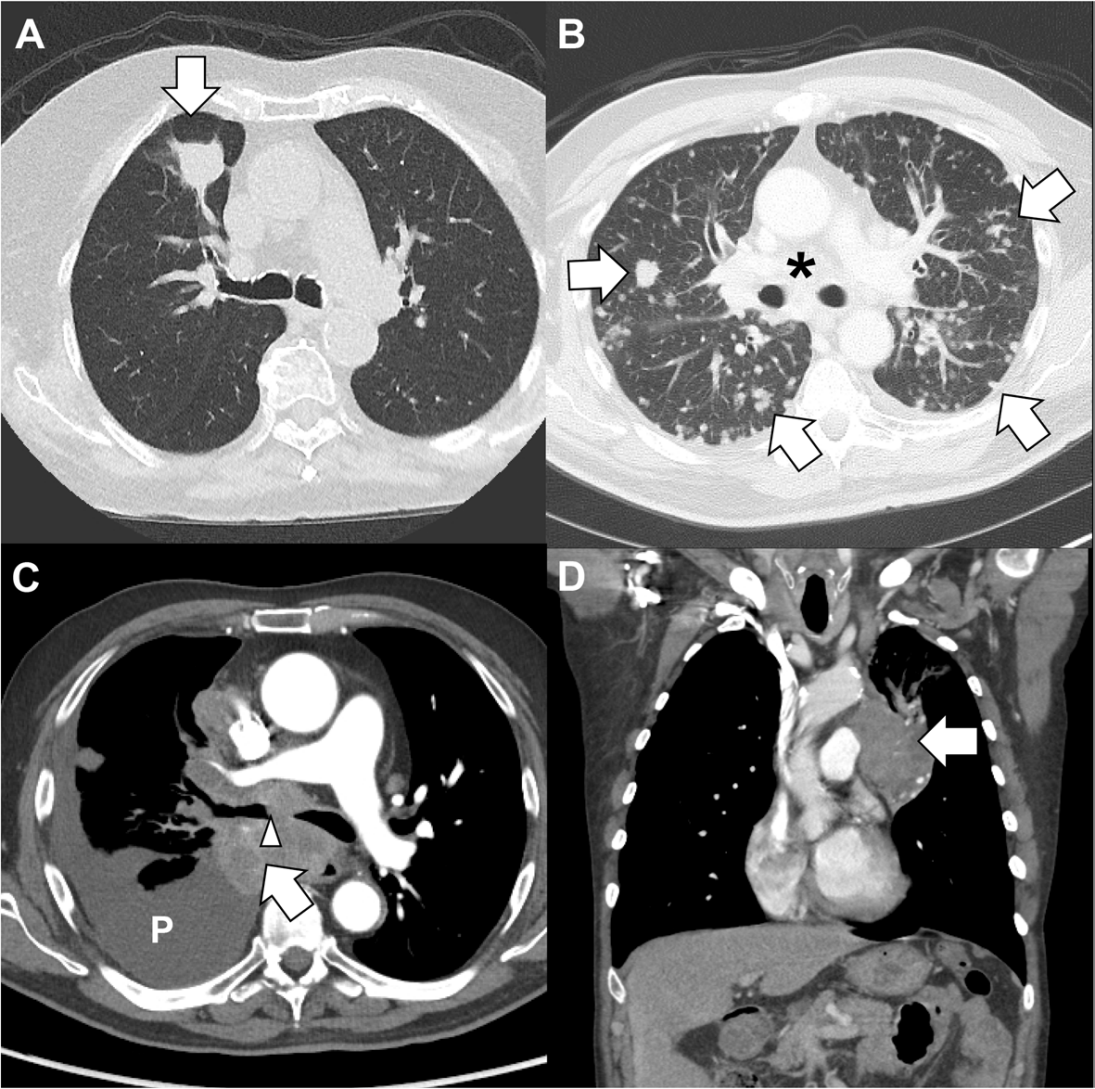
ED presentations of lung cancer. **A** Elderly woman who is a never-smoker presents with cough and fatigue. Imaging revealed a 1.8-cm right upper lobe lung nodule (arrow) without hilar or mediastinal lymphadenopathy. She underwent lobectomy (stage IA, pT1c pN0 cM0). **B** Middle-aged man with metastatic adenocarcinoma of the lung was sent for anemia and fatigue. Imaging reveals innumerable pulmonary nodules (arrows) with mediastinal, bilateral hilar, and subcarinal lymphadenopathy (asterisk). **C** Middle-aged man presents with hemoptysis and dyspnea. Imaging reveals endobronchial disease in the right main bronchus at the level of the carina (arrowhead), moderate pleural effusion (P), and lung mass (arrow). **D** Middle-aged man with chronic myeloid leukemia on imatinib transferred for airway exacerbation. Imaging incidentally revealed a left upper lobe mass (arrow) contiguous with mediastinal and hilar lymphadenopathy consistent with primary lung malignancy

Lung cancer staging may be performed radiographically with multiple modalities (Fig. 3). Measuring tumor size with chest CT remains the first-line standard [98]. Due to its high sensitivity (77.4% CI 65.3–86.1) and specificity (90.1% CI 85.3–93.5) for mediastinal nodal metastasis detection according to the Cochrane Review of 2014, ^18^F-fluorodeoxyglucose (FDG) positron emission tomography (PET)/CT can be useful in differentiating the nodal stage [99]. FDG PET/CT imaging better visualizes distant extra-thoracic metastases with extremely high sensitivity and specificity [100]. PET/CT also reduces the number of unnecessary thoracotomies by detecting increased mediastinal nodal metastasis detection and improves sensitivity in preoperative lung cancer staging [101].

After excluding the presence of distant metastases, clinicians must determine the extent of intrathoracic nodal metastasis. With primary peripheral pulmonary tumors < 3 cm with a negative FDG PET/CT, mediastinal sampling may not be necessary prior to surgical intervention [102]. Despite radiographic imaging, accurate invasive mediastinal staging must be obtained in order to prevent harm from understaging or overstaging of disease [103]. Depending on the availability of specialists, mediastinal lymph nodes may be sampled by interventional pulmonologists or radiologists, surgeons, or gastroenterologists. Procedures performed include endobronchial ultrasound with transbronchial needle aspiration (EBUS-TBNA), endoscopic ultrasound (EUS), CT-guided biopsy, mediastinoscopy, and mediastinotomy. Although mediastinoscopy remains the procedure with the highest negative predictive value for nodal disease, EBUS-TBNA is a minimally invasive procedure that has almost equivalent diagnostic accuracy and therefore is the standard of care when lymph nodes are accessible by this route [104].

### Multi-disciplinary care

Cancer care is multi-disciplinary. Screening, staging, diagnosing, and treating lung cancer involve many different specialties including primary care, thoracic surgery, oncology, pulmonology, emergency medicine, radiology, interventional radiology, palliative care, pathology, specialty nursing, and radiation oncology [105]. Although much of cancer staging and the correlated treatment has been established in guidelines, many alternatives and adjunctive therapy can be added by multidisciplinary teams. In 2010, the incorporation of palliative care teams in patients with metastatic NSCLC improved quality of life and survival [106]. Case-by-case, a multidisciplinary team can add treatment options including management of endobronchial disease, enrollment in clinical trials, addition of radiotherapy, and management of complications related to therapy and disease progression [107].

### Treatment

Treatment of lung cancer is currently limited by the clinical stage and performance status of the patient. Video-assisted thoracoscopic surgery (VATS) or thoracotomy is the standard of care for patients with stage I or II lung cancer (147). Those with stage I cancer and contraindications to surgery may benefit from stereotactic body radiotherapy (SBRT) as an alternative [108]. More advanced nodal staging shifts the focus of treatment to chemotherapy and immunotherapy. In NSCLC, first-line chemotherapy with a platinum-based doublet includes carboplatin or cisplatin in combination with taxanes, gemcitabine, vinorelbine, or pemetrexed [109]. The addition of bevacizumab, a monoclonal antibody against vascular endothelin growth factor (VEGF), is sometimes added with initial cycles of chemotherapy [110]. In those with non-squamous variants, cisplatin with pemetrexed had increased survival when compared with cisplatin and gemcitabine [111]. After 4–6 cycle induction with cisplatin/pemetrexed, maintenance pemetrexed has been shown to improve progression-free and overall survival [112]. Alternatively, SCLC treatment involves platinum therapy plus etoposide as the first-line chemotherapy [113].

Cancer immunotherapy activates the immune system to aid in the detection and destruction of tumor cells. Immune checkpoint inhibitors (ICI) are now commonplace for the treatment of lung cancer and include programmed cell death ligand 1 (PD-L1) inhibitors like atezolizumab or durvalumab and programmed cell death 1 (PD-1) inhibitors like nivolumab and pembrolizumab [114, 115]. Chemotherapy and immunotherapy combinations in SCLC have shown increased progression-free survival [116, 117].

When developing a treatment plan, tumor biomarkers are sent to aid the selection of immunotherapy. Most recently, advanced immunohistochemical and molecular profiling can assist with targeted therapy for lung cancer. Actionable mutations have been identified (KRAS, EGFR, ALK, ROS1, and others), and subsequent specific molecular inhibitors have been developed (sotorasib, osimertinib, ceritinib, crizotinib) for the treatment of these cancers [118].

Newer evidence has led to a discussion of metastases as a spectrum from limited cancer to diffusely metastatic. In the center of the spectrum is an oligometastatic disease which is characterized by less than 3–5 metastases [119]. In this rare subset, multidisciplinary surgical and radiation ablative adjunct therapies in combination with chemotherapy and immunotherapy may prolong survival.

Emergency physicians are familiar with managing airway emergencies and addressing pulmonary symptoms in critically ill patients, but symptom palliation in terminal disease requires a different perspective. Although heroic measures may not be indicated, ameliorating symptoms of breathlessness and dyspnea are paramount. The sensation of dyspnea can be multifactorial and not necessarily related to hypoxia. The American Society of Clinical Oncology guidelines recommend a hierarchical approach to dyspnea in advanced cancer including palliative care consultation, non-pharmacologic interventions (fanning directed toward the cheek, supplemental oxygen, high-flow oxygen, non-invasive positive pressure ventilation), and pharmacologic interventions (systemic opioids, short-acting benzodiazepines, systemic corticosteroids, bronchodilators) [120].

## Other presentations of lung cancer

### Manifestations of disease

**Fig. 4:**
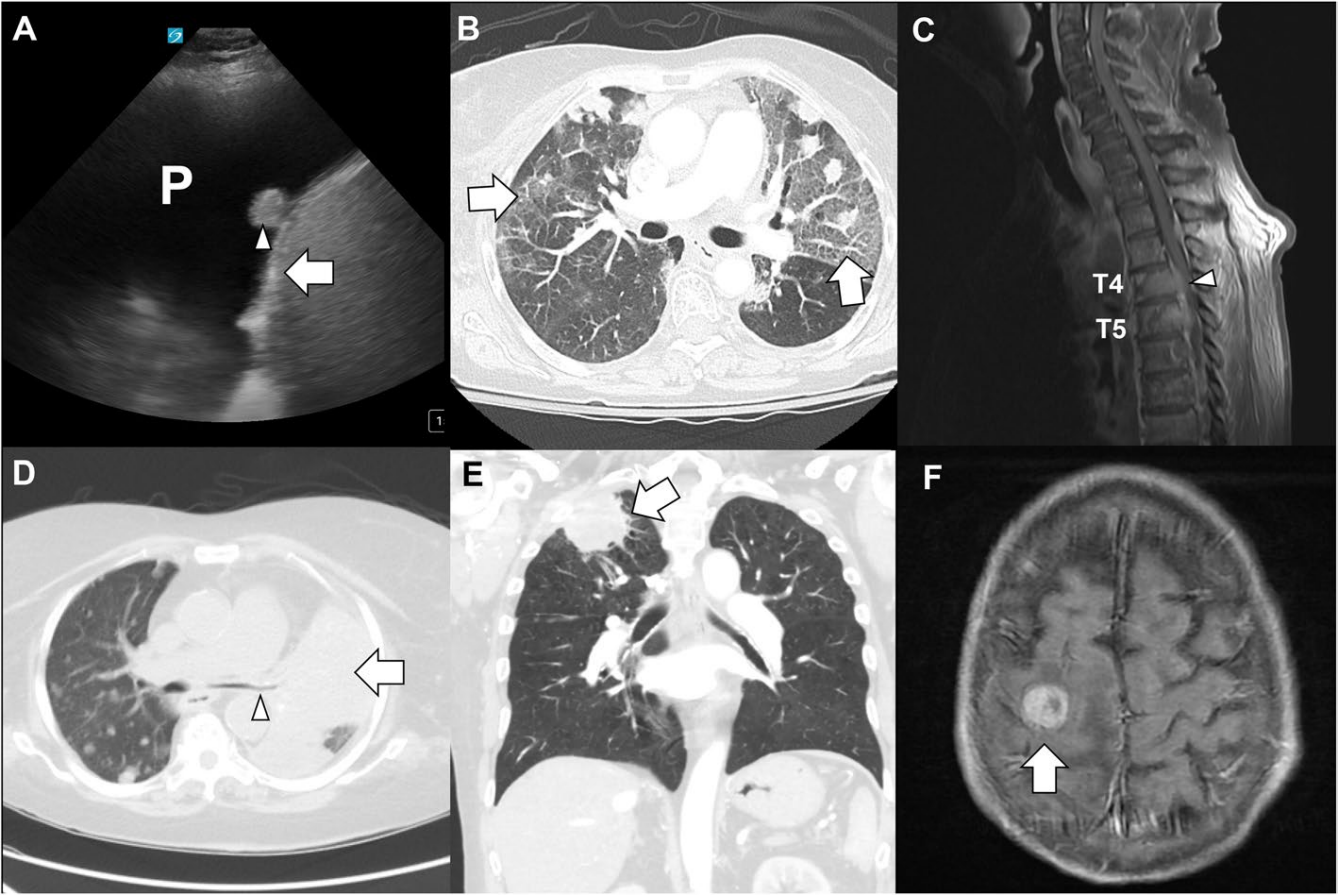
Manifestations of disease. **A** Ultrasound image of suspected malignant pleural effusion (P) with nodule (arrowhead) on the diaphragm (arrow). **B** Lung cancer on immune checkpoint inhibitor with multiple nodules and ground glass infiltrates (arrows, left > right) concerning immune-mediated pneumonitis. **C** Extensive small cell lung cancer with metastatic disease to spine (T4, T5) and extension of epidural tumor (arrowhead). **D** Left lung mass (arrow) with extrinsic compression (arrowhead) of the left mainstem bronchus. **E** Elderly aged man with a right upper lobe lung mass (arrow) in the apex consistent with a Pancoast tumor (mass that originates in the superior sulcus of the lung apex which may present atypically with shoulder pain, Horner syndrome, and superior vena cava syndrome) presenting with right shoulder pain and cough. **F** Middle-aged man with metastatic poorly differentiated lung cancer presents to the emergency center after fall. Imaging reveals a 1.8-cm right posterior frontal mass (arrow) with local edema

Intrathoracic disease results from tumor involvement or spread including extrinsic compression of the tracheobronchial tree, malignant airway obstruction or endobronchial disease, vasculature (superior vena cava syndrome), diaphragmatic or vocal cord dysfunction, and pleural effusion. These may contribute to symptoms of cough, hemoptysis, chest pain, and dyspnea. Extrathoracic manifestations of disease depend on the area of involvement and if metastatic can involve the central nervous system, bone, adrenal glands, or liver (Fig. 4). Imaging reveals the lesions and helps interventionalists plan treatment.

### Therapy-related sequelae

Medical, surgical, and radiation treatments of lung cancer have the risk of therapy-related injury. Postoperative surgical complications, though rare, include atelectasis, infection, pulmonary embolism, nerve injury, respiratory failure, bronchopleural fistula, hemothorax, and cardiac arrhythmias [121]. Radiation can lead to acute pneumonitis, chronic pulmonary fibrosis, dermatitis, esophagitis, radiation myelitis, and secondary malignancy [122].

ICI treatments cause autoimmune-like complications called immune-related adverse events (irAEs). irAEs can affect any organ system and may cause thyroid dysfunction, diabetes, dermatitis, myocarditis, myositis, pneumonitis (Fig. 4), colitis, hypophysitis, hepatitis, myasthenia gravis, and many others [123]. When present, irAEs frequently affect multiple organ systems. Management of irAEs may include discontinuing ICI therapy temporarily or permanently and systemic corticosteroid administration. Most cases improve or resolve with steroid treatment, but steroid-refractory cases may be treated with other immunosuppressive agents [124].

Both radiation and ICI sequelae can be long-term and affect patients long after the cancer is cured.

## Conclusion

ED providers must be aware of various lung cancer presentations. These include referring newly discovered or suspected cases to prompt a workup for definitive diagnosis. In addition, they must be able to discern symptoms of malignant disease from therapy-related sequelae. As the landscape of anti-neoplastic treatments evolves and includes immune-mediated and targeted therapies, patients with lung cancer may survive longer. The ED has a potentially unique role in identifying those at risk of lung cancer especially in underserved populations and referring them to cancer prevention (smoking cessation) and screening programs. Therefore, with a knowledge base regarding the newest screening guidelines, current imaging findings, updated cancer staging, medical and surgical treatment options, multidisciplinary care, and therapy-related sequelae in the community, the ED becomes a key mediator of multidisciplinary care for patients at risk for and living with lung cancer. As lung cancer detection and treatment advances, so the importance of the ED in lung cancer evolves as well.

## Data Availability

Not applicable.

## Abbreviations

AJCC: American Joint Committee on Cancer
CMS: Centers for Medicare and Medicaid Services
CT: Computed tomography
EBUS-TBNA: Endobronchial ultrasound with transbronchial needle aspiration
EGFR: Epidermal growth factor receptor
EUS: Endoscopic ultrasound
FDG: 18F-Fluorodeoxyglucose
HIV: Human immunodeficiency virus
ICI: Immune checkpoint inhibitors
irAEs: Immune-related adverse events
LDCT: Low-dose computed tomography
Lung-RADS®: Lung CT Screening Reporting & Data System
NCCN: National Comprehensive Cancer Network
NEDS: Nationwide Emergency Department Sample
NELSON: Nederlands-Leuvens Longkanker Screenings Onderzoek
NLST: National Lung Screening Trial
NSCLC: Non-small cell lung cancer
PCP: Primary care provider
PD-1: Programmed cell death 1
PD-L1: Programmed cell death ligand 1
PET/CT: Positron emission tomography/CT
SBRT: Stereotactic body radiotherapy
SCLC: Small cell lung cancer
USPSTF: US Preventative Services Task Force
VATS: Video-assisted thoracoscopic surgery
VEGF: Vascular endothelin growth factor
